# Timp1 interacts with beta-1 integrin and CD63 along melanoma genesis and confers *anoikis* resistance by activating PI3-K signaling pathway independently of Akt phosphorylation

**DOI:** 10.1186/1476-4598-12-22

**Published:** 2013-03-25

**Authors:** Mariana Toricelli, Fabiana HM Melo, Giovani B Peres, Débora CP Silva, Miriam G Jasiulionis

**Affiliations:** 1 Pharmacology Department, Universidade Federal de São Paulo, São Paulo, Brazil; 2 Microbiology, Immunology and Parasitology Department, Universidade Federal de São Paulo, São Paulo, Brazil; 3 Biochemistry Department, Universidade Federal de São Paulo, São Paulo, Brazil; 4 Ludwig Institute for Cancer Research, São Paulo, Brazil

**Keywords:** *Timp1*, Beta1-integrin, CD63, PI3-K pathway, *Anoikis*, Melanoma, Malignant transformation

## Abstract

**Background:**

*Anoikis* resistance is one of the abilities acquired along tumor progression. This characteristic is associated with metastasis development, since tumorigenic cells must survive independently of cell-matrix interactions in this process. In our laboratory, it was developed a murine melanocyte malignant transformation model associated with a sustained stressful condition. After subjecting melan-a melanocytes to 1, 2, 3 and 4 cycles of anchorage impediment, *anoikis* resistant cells were established and named 1C, 2C, 3C and 4C, respectively. These cells showed altered morphology and PMA independent cell growth, but were not tumorigenic, corresponding to pre-malignant cells. After limiting dilution of 4C pre-malignant cells, melanoma cell lines with different characteristics were obtained. Previous data from our group showed that increased Timp1 expression correlated with *anoikis*-resistant phenotype. Timp1 was shown to confer anchorage-independent growth capability to melan-a melanocytes and render melanoma cells more aggressive when injected into mice. However, the mechanisms involved in *anoikis* regulation by Timp1 in tumorigenic cells are not clear yet.

**Methods:**

The β1-integrin and Timp1 expression were evaluated by Western blotting and CD63 protein expression by flow cytometry using specific antibodies. To analyze the interaction among Timp1, CD63 and β1-integrin, immunoprecipitation assays were performed, *anoikis* resistance capability was evaluated in the presence or not of the PI3-K inhibitors, Wortmannin and LY294002. Relative expression of *TIMP1* and *CD63* in human metastatic melanoma cells was analyzed by real time PCR.

**Results:**

Differential association among Timp1, CD63 and β1-integrins was observed in melan-a melanocytes, 4C pre-malignant melanocytes and 4C11- and 4C11+ melanoma cells. Timp1 present in conditioned medium of melanoma cells rendered melan-a melanocytes *anoikis*-resistant through PI3-K signaling pathway independently of Akt activation. In human melanoma cell lines, in which TIMP1 and beta-1 integrin were also found to be interacting, *TIMP1* and *CD63* levels together was shown to correlate significantly with colony formation capacity.

**Conclusions:**

Our results show that Timp1 is assembled in a supramolecular complex containing CD63 and β1-integrins along melanoma genesis and confers *anoikis* resistance by activating PI3-K signaling pathway, independently of Akt phosphorylation. In addition, our data point *TIMP1*, mainly together with *CD63*, as a potential biomarker of melanoma.

## Introduction

Cutaneous melanoma originates from malignant transformation of melanocytes. As in the case of most cancers, it is believed that genetic and environmental factors contribute to development of melanoma. Although melanoma is the less frequent skin cancer (about 4%), it is responsible for most deaths (approximately 80%) due its high incidence of metastases [1,2]. Cell-extracellular matrix interactions are a key factor for melanocyte homeostasis and disruption of such interactions has adverse effects on cell survival, driving a specific type of apoptosis known as *anoikis*. This kind of apoptosis following loss of cell anchorage is important for development, tissue homeostasis and several diseases, including cancer [3]. *Anoikis* has a particular importance in tumor progression, since the acquisition of an *anoikis*-resistant phenotype is one of the critical steps acquired during malignant transformation [4]. In animal models, *anoikis*-resistant tumor cells are associated with high incidence of metastatic lesions and increased cell survival in blood [5-8]. Furthermore, this property observed in these cells when cultured *in vitro* can be correlated to its oncogenic potential found *in vivo*[9].

A previous work of our group showed that the acquisition of *anoikis*-resistant phenotype is associated with melanocyte malignant transformation [10]. Oba-Shinjo and colleagues (2006) demonstrated that sequential cycles of anchorage blockade resulted in malignant transformation of melan-a murine melanocytes [10]. As expected for a non-tumorigenic immortalized cell line, most melan-a cells underwent *anoikis* when maintained in suspension. However, small spheroids were observed after maintaining melan-a cells in suspension for 96 hours. Melan-a cells resistant to *anoikis* were cultured in adherent conditions and subjected to further deadhesion cycles. Cell lines were established after subjecting melan-a melanocytes to 1, 2, 3 and 4 cycles of anchorage blockade (1C, 2C, 3C and 4C cell lines, respectively). These cell lines were non-tumorigenic, but showed morphological changes, increased *anoikis*-resistant phenotype and alterations in the expression of different molecules [10], being considered pre-malignant cells. Pre-malignant 4C melanocytes were subjected to a new cycle of anchorage blockade and the spheroids formed were submitted to a limiting dilution. All clones, randomly selected, were tumorigenic when subcutaneously inoculated into syngeneic mice. In this way, different melanoma cell lines (i.e. 4C11- and 4C11+) were established and showed differences in pigmentation, aggregation, *in vitro* and *in vivo* proliferation and ability to metastasize [10].

TIMP1 is a member of the family of matrix metalloproteinase (MMP) inhibitors that is composed of four members (TIMP1, TIMP2, TIMP3 and TIMP4). As the name suggests, the main function of TIMP1 is to inhibit extracellular matrix degradation mediated by MMPs. However, TIMP1 may interact with other proteins and regulate biological processes such as cell growth, apoptosis and differentiation, independently of its metalloproteinase inhibitory activity [11-15]. In this context, CD63, a member of the tetraspanin family, was first identified as an antigen associated with early stages of human melanoma and as a binding partner of TIMP1 on the cell surface [15]. Jung and coworkers [15] showed a correlation between the expression of TIMP1 and the level of active β1-integrin on the surface of epithelial breast cells, independent of cell adhesion, and showed interaction among TIMP1, CD63 and β1-integrin. Inhibition of CD63 expression was able to effectively reduce TIMP1 binding on the cell surface and its co-localization with β1-integrins. Besides that, β1-integrins activation, signaling survival activation and inhibition of apoptosis mediated by TIMP1 was abrogated. The mammals’ integrin family contains 18 α-subunits and eight β-subunits that form 24 distinct receptors with specific tissue distribution that appear to have specific and non-redundant functions as shown by their specificity for ECM ligands and knockout mouse phenotypes [16]. The primary function of integrin family is to mediate cell-cell and cell-matrix adhesion. Furthermore, the binding of ECM components to integrins leads to the recruitment of numerous adaptor and signaling proteins to the cytoplasmic tails of the integrin β-subunits, forming adhesion protein complexes that initiate signaling cascades promoting cell polarity, motility, differentiation, proliferation and survival [16]. β1-integrins are expressed in a wide variety of tissues and different cell types throughout the body. They are critical in the induction and maintenance of cell differentiation and are involved in various physiological functions and in tissue homeostasis [16].

Previously, we reported increased *Timp1* expression along melanoma genesis and a tight correlation between *Timp1* expression and its promoter methylation during melanocytes malignant transformation [17]. It was demonstrated that Timp1 confers resistance to *anoikis*, since melanocytes overexpressing Timp1 become able to resist to *anoikis* and form colonies in soft-agar. Moreover, melanoma cells overexpressing Timp1 acquire increased capacity to grow and metastasize *in vivo*[17]. However, the signaling pathway induced by Timp1 to protect melanoma cells from apoptosis is still unknown.

The phosphoinositide 3-kinase (PI3K) signaling pathway activation regulates fundamental cellular functions such as transcription, growth, differentiation and survival, mostly through AKT phosphorylation [18]. PI3K/AKT signaling is associated with the disruption in the balance between cell proliferation and apoptosis, and has been related with the development of diseases such as cancer, autoimmunity and diabetes mellitus [18].

In this work, we have shown for the first time the assembly of a supramolecular complex containing Timp1, CD63 and β1-integrins at the cell surface in melanoma cells, and its involvement in the acquisition of an *anoikis*-resistant phenotype through PI3K signaling pathway independently of Akt activation. In addition, our data points TIMP1 as a biomarker of human melanoma.

## Material and methods

### Cell culture

The non-tumorigenic melan-a melanocyte lineage [19] was cultured at 37°C in humidified 95% air-5% CO_2_ in RPMI pH 6.9 supplemented with 5% fetal bovine serum (Invitrogen, Scotland, UK), 200 nM 12-phorbol-13-myristate acetate (PMA; Calbiochem, Darmstadt, Germany), 100 U/ml penicillin, and 100 U/ml streptomycin (Invitrogen, Grand Island, NY). PMA activates protein kinase C, and is required for melanocytes to survival and proliferate in culture [19]. Pre-malignant 4C melanocyte lineage, non-metastatic 4C11- and metastatic 4C11+ melanoma cell lines were cultured as melan-a cells, but in the absence of PMA, since they lost the requirement of this factor to grow. Stably Timp1-overexpressing melan-a (MaT1S) and the control MaGFP were cultured in the same conditions described above in the presence of PMA. The plasmid construction and transfection was previously described [17]. Primary human melanocytes MP#2, kindly provided by Dr. Silvya Stuchi Maria-Engler (Faculdade de Ciências Farmacêuticas, Universidade de São Paulo), was cultured at 37°C in humidified 95% air-5% CO_2_ in Cascade growth medium 254 (Gibco, Grand Island, NY) supplemented with 200 μM CaCl_2_ and 1% HGMS (human growth melanocyte supplement). The patient-derived metastatic melanoma cells Mel2, Mel3, Mel4, Mel11, Mel14, Mel21, Mel25, Mel28 and Mel33 kindly provided by Dr. Débora C.P. Silva (Ludwig Institute for Cancer Research, São Paulo), were cultured at 37°C in humidified 95% air-5% CO_2_ in RPMI pH 7.2 supplemented with 15% fetal bovine serum (Invitrogen, Scotland, UK), 1 mM sodium piruvate, 2 mM L-glutamine and antibiotics (Gibco, Grand Island, NY).

### mRNA expression analysis

RNA was isolated from cell monolayers (MP#2, Mel2, Mel3, Mel11, Mel14, Mel21, Mel25, Mel33, Melan-a, 4C, 4C11- and 4C11+) using TRIzol® (Invitrogen, Carlsbad, CA). cDNA was prepared from 1 μg of RNA using random hexamer primers and OligodT (Superscript III first-strand synthesis system for RT-PCR, Invitrogen). For *TIMP1* and *CD63* mRNA expression analysis, quantitative real-time PCR were performed using a Corbett Rotor-Gene 6000 detection system® with a Fast Rotor-Gene SYBR Green PCR Master Mix® (Qiagen, Dusseldorf, Germany). Specific primers were used as follows: human *TIMP1* (sense: 5’ ATG TTA CTG TGG GCT GTG G 3’, antisense: 5’ CGA CAA AAG CAA TTC CAA GGG 3’), human *CD63* (sense: 5’ CCC TTG GAA TTG CTT TTG TCG 3’, antisense: 5’ CGT AGC CAC TTC TGA TAC TCT TC 3’), human *GAPDH* (sense: 5’ GTC TTC CCC TCC ATC GTG 3’, antisense: 5’ GTA CTT CAG GGT GAG GAT GC 3’), mouse *timp1* (sense: 5’ GCT AAA AGG ATT CAA GGC 3’, antisense: 5’ GCA CAA GCC TAG ATT CCG 3’), and mouse *gapdh* (sense: 5’ CCA TGG AGA AGG CTG GGG 3’ antisense: 5’ CAA AGT GTC ATG GAT GAC A 5’).

### Western Blotting

Subconfluent cell cultures were trypsinized, washed with PBS and membrane-enriched protein extracts were prepared using cold lysis buffer (1% Triton X-100 in 150 mM NaCl and 50 mM Tris pH 7.4, containing 5 mM EDTA, CHAPS 1%, 2 g/mL aprotinin and 1 mM PMSF), kept for 15 minutes on ice, followed by centrifugation at 10.000 rpm for 15 minutes at 4°C. Alternatively, MaT1S and MaGFP cells submitted for 1 h (D1h), 3 h (D3h), 5 h (D5h) and 24 h (D24h) were washed and cytoplasmic protein extracts were carried out using cold lysis buffer (0.5% NP40 in 100 mM NaCl and 50 mM Tris pH7.4, containing 50 mM NaF, 1 mM NaVO_4_, 2 g/mL aprotinin and 1 mM PMSF), kept for 15 minutes on ice, followed by centrifugation at 10.000 rpm for 15 minutes at 4°C. The supernatant was collected and protein concentration was measured by Bio-Rad protein assay dye reagent concentrate (Bio-Rad, Hercules, CA). Equivalent amounts of protein (50 μg) were denaturated in SDS sample buffer (240 mM Tris–HCl pH 6.8, 0.8% SDS, 200 mM beta- mercaptoethanol, 40% glycerol and 0.02% bromophenol blue) for 5 minutes, then separated by electrophoresis in SDS-polyacrylamide gels and transferred to a polyvinylidene difluoride membrane (Amersham, Piscataway, NJ). After protein transfer, the membranes were blocked with 5% non-fat dry milk in PBS (10mM phosphate buffer pH 7.2, 150 mM NaCl), incubated with the indicated antibodies: goat polyclonal anti-Timp1 (R&D), rabbit polyclonal anti-β1-integrin (Calbiochem), rabbit polyclonal anti-CD63 (Santa Cruz), rabbit polyclonal anti-phospho Akt (Cell Signaling) and rabbit polyclonal Akt (Cell Signaling) overnight at 4°C, and the signal was detected using horseradish peroxidase–conjugated anti–immunoglobulin G antibody (KPL; Gaithersburg, MD) followed by development using chemiluminescence substrate (SuperSignal West Pico Chemiluminescent Substrate; Pierce Chemical, Rockford, IL).

### Metalloprotease activity

Gelatinase activity was determined by zymography, as previously described [20,21]. In brief, 48 hours conditioned media (10 μg) were submitted to 10% polyacrilamide gel electrophoresis with 1% gelatin as substrate (30 mA per gel, in ice bath). After run, gels were washed four times in 2% Triton X-100, 5 min each, in order to remove SDS and renaturate enzymes. Gels were then incubated overnight at 37°C in 50 mM Tris–HCl buffer pH 8.2, containing 5 mM CaCl_2_ and 0.5 μM ZnCl_2_ (incubation buffer). Gels were stained with 0.5% Coomasie Brilliant Blue R-250 in 30% methanol, 10% acetic acid for 30 min, and then properly destained until clear (degraded) bands could be seen. Zymograms were scanned with Epson Expression 1680 flatbed scanner (Epson America, Inc., Long Beach, CA, USA), and densitometric analysis was performed with TotalLab Quant v11 (TotalLab Ltd., Newcastle, UK). Experiments were always performed in quadruplicates.

### Colony formation assay

MaGFP, MaT1S, MP#2, Mel2, Mel3, Mel4, Mel11, Mel 25, Mel33 cells (1×10^2^) were cultured in adherent conditions in complete medium on 6-well plates to allow colony formation. After seven days, colonies were washed with PBS, fixed in 3.7% (v/v) formaldehyde for 15 minutes, stained with 1% Toluidine blue 1% Borax for 5 minutes and washed with water. For quantification of survival cells, the staining was dissolved in 1% SDS and the absorbance at 570 nm was evaluated using an ELISA microplate reader.

### Flow cytometry analysis

Cultured cells were harvested with trypsin. After trypsin inactivation, cells were washed with PBS containing 1% BSA and incubated with anti-CD63 (Santa Cruz) diluted in 1% BSA in PBS for 1 hour under agitation at 4°C. After incubation, cells were washed with PBS and incubated with secondary antibody anti-IgG Alexa-488 (Molecular Probes) for 45 minutes under agitation at room temperature. After three washes with PBS containing 0.1% BSA, cells were analyzed in a flow cytometer (FACScalibur, Becton Dickinson).

### Co-immunoprecipitation assays

Extracts from murine melan-a, 4C, 4C11- and 4C11+ cell lines and human MP#2, Mel2, Mel3, Mel14 cell lines were pre-cleared with 50 μl of G protein-coupled to agarose beads (Gibco BRL, Gaitherburg, MD) for 2 hours at 4°C under agitation. The suspension was centrifuged at 10.000 rpm for 15 minutes at 4°C and the collected supernatant was incubated overnight at 4°C with specific antibodies against CD63 (Santa Cruz), β1-integrin (Calbiochem) and TIMP1 (Santa Cruz). The suspension was incubated with G protein-agarose for 4h at 4°C under agitation and centrifuged at 6000 rpm for 10 seconds at 4°C. The beads were collected and washed three times for 10 minutes at 4°C with wash buffer A (10 mM Tris pH 8.0, 150 mM NaCl, 0.1% Triton X-100) and two times with wash buffer B (10 mMTris pH 6.8). Beads were added to 60 μL of reducing sample buffer, which were boiled for 5 min, separated by electrophoresis on polyacrylamide-SDS 10% and 15%, transferred to PVDF membrane and incubated with antibodies of interest.

### Conditioned medium

Melan-a and 4C11+ cell lines (1×10^6^) were maintained in RMPI medium pH 6.9 with 0.5% fetal bovine serum for 48 hours. After 48 hours, the supernatant was collected and centrifuged at 2000 rpm for 3 minutes and used in cell survival experiments.

### *Anoikis* resistance assay

This assay was performed in two ways. First, to evaluate the relative number of surviving cells (adherent and suspended cells) after deadhesion, MTT (3-(4,5-Dimethylthiazol-2-yl)-2,5-diphenyltetrazolium bromide) assay was performed. For this, adherent melan-a cells were harvested by mild trypsin treatment and 1×10^5^ cells were cultured per mL on 1% (w/v) agarose-coated plates for 96 hours (D96h) in the absence or presence of melan-a or 4C11+ conditioned medium and in the absence or presence of 20 μL of Timp1-neutralizing antibody (R&D) in fresh medium containing 5% (v/v) FBS. After 96 h in suspension, cells were collected, transferred to 96-well plate and incubated with MTT. To ensure that all viable cells would be analyzed, the plate was centrifuged, cells were lysed with isopropanol and the absorbance at 620 nm was recorded in each well using an ELISA microplate reader. This experiment was performed in biological triplicate. Statistical analysis were made using Student's T test for unpaired samples. Second, to evaluate the relative number of cells able to form colonies after submitted to anchorage impediment, melan-a, 4C11-, 4C11+, MaT1S and MaGFP cells were cultured in complete medium for 24 hours on 6-well plates. These cells were harvested by mild trypsin treatment and 2.5×10^3^ cells per mL were cultured on 1% (w/v) agarose-coated plates for 96 hours (D96h) in the absence or presence of melan-a or 4C11+ conditioned medium, Timp1 neutralizing antibody (R&D), 0.5 μM Wortmannin, a PI3-K inhibitor, (Calbiochem, Darmstadt, Germany) or 2.5 μM LY294002, a PI3-K specific inhibitor (Sigma) in fresh medium containing 0.5% (v/v) FBS at 37°C in 5% CO_2_. The cells were collected by centrifugation, seeded on 60 mm-dishes and grown for five days to allow colony formation. Colonies were washed with PBS, fixed in 3.7% (v/v) formaldehyde for 15 minutes, stained with 1% Toluidine blue for 5 minutes and washed with water. For quantification of survival cells, the staining was lysed in 1% SDS and absorbance at 570 nm was evaluated using an ELISA microplate reader.

### Statistical analysis

All tests were conducted in biological triplicate. The results were organized into a database using the statistical program GraphPad Prism 5 (GraphPad Software, Inc. - California, USA). The level of significance utilized was p<0.05. The statistical tests performed were: Student's T test for unpaired samples, One-way ANOVA followed by Tukey's multiple comparison tests and Spearman's Rank Correlation.

## Results

### Increased levels of membrane-bound and secreted Timp1 in cells representing melanoma progression

**Figure 1:**
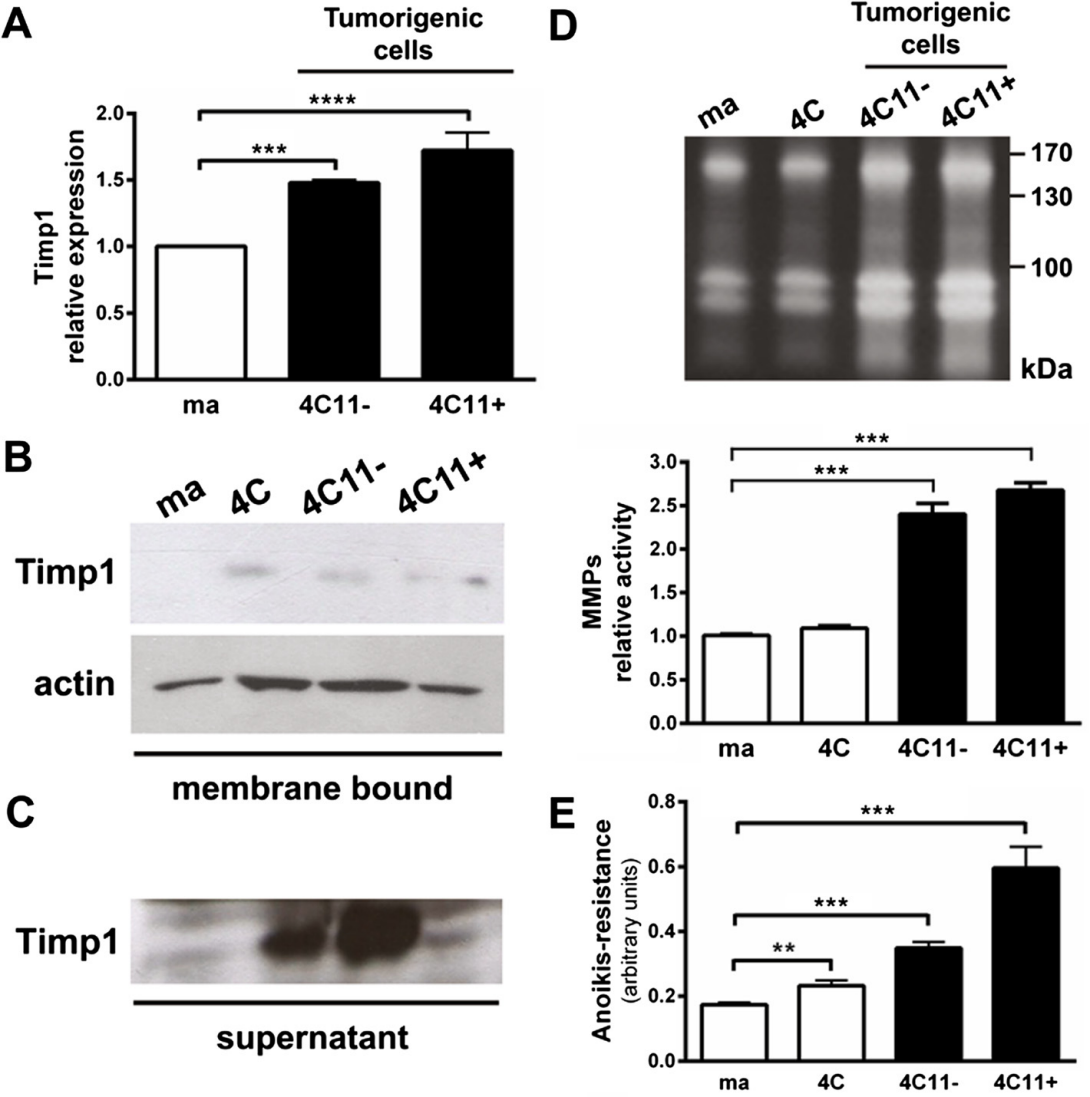
**Increased association of Timp1 on the cell surface along melanoma progression. A.** mRNA levels of *Timp1* were determined by real time PCR. **B.** Membrane-enriched extracts were separated on 15% polyacrylamide gel SDS/PAGE and transferred to a PVDF membrane. The membrane was incubated with polyclonal antibody specific to Timp1. The constitutive expression of β-actin was used as endogenous control. **C.** The supernatants of cell cultures were collected and lyophilized. The presence of soluble Timp1 was identified by Western Blotting. **D.** Conditioned media (10 μg) from melan-a, 4C, 4C11- and 4C11+ cell lines were evaluated for MMPs activity. **E.** The same cell lines were maintained in suspension for 96 hours and viable cells were estimated using MTT. Experiments were always performed in quadruplicates. ma: murine non-tumorigenic melanocyte lineage; 4C: pre-malignant melanocytes; 4C11-: non-metastatic melanoma cells; 4C11+: metastatic melanoma cells. **p < 0.01, ***p < 0.001, ****p < 0.0001.

Previous data from our laboratory have shown significant and progressive increase in the expression of *timp1* in cells representing different phases of melanoma progression. This increase was accompanied by *anoikis* resistance and more aggressive phenotype *in vivo*[17]. In agreement with our previous data, a progressive increase of Timp1 mRNA expression was found in 4C11- and 4C11+ melanoma cell lines (Figure 1A), as described for other melanoma cell lines from our model [17]. To analyze the Timp1 protein expression and especially its association with cell surface, membrane-enriched extracts of melan-a, 4C, 4C11- and 4C11+ cell lines were separated by SDS/PAGE. Timp1 protein expression was observed in all cell lines (4C, 4C11- and 4C11+) derived from anchorage impediment of melan-a melanocytes (Figure 1B). Soluble Timp1 was found in the conditioned media from pre-malignant 4C, and 4C11- and 4C11+ melanoma cell lines, but not in that from non-tumorigenic melan-a melanocytes (Figure 1C). Although the most known function of Timp1 is to inhibit matrix metalloproteinases, no correlation between Timp1 expression and MMPs activity was observed in our model, since Timp1-expressing melanoma cells presented high MMP activity (Figure 1D). Figure 1E shows that cell lines representing melanoma progression and expressing high levels of Timp1 present increased capability to form colonies compared to melan-a melanocytes.

### Timp1 associates with β1-integrin and CD63 along melan-a melanocyte malignant transformation

**Figure 2:**
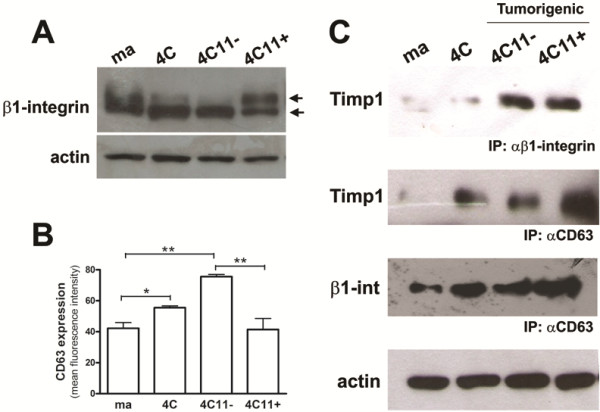
**A tight Timp1/CD63/β1-integrin complex is formed only in tumorigenic cell lines. A.** β1-integrin expression pattern was analyzed in murine melan-a, 4C, 4C11- and 4C11+ cell lines by Western blotting using a specific polyclonal antibody. Note the shift of β1-integrin eletrophoretic migration in the metastatic 4C11+ melanoma cell line. The same membrane was reprobed with anti-actin antibody as an endogenous control. **B.** CD63 protein expression was evaluated by flow cytometry using antibody specific. **C.** Membrane-enriched protein extracts from melan-a, 4C, 4C11-, and 4C11+ cell lines were immunoprecipitated with anti-CD63 and anti-β1-integrin and analyzed by Western blotting with anti-Timp1 and anti-β1-integrin. ma: non-tumorigenic melanocytes melan-a; 4C: pre-malignant melanocytes; 4C11-: non-metastatic melanoma cells; 4C11+: metastatic melanoma cells. *p < 0.05, **p < 0.01.

Based on its importance and well-established role, β1-integrin expression profile was first investigated in our model by Western Blot. A differential migration shift was observed in the bands corresponding to β1-integrin along melanoma genesis (Figure 2A). Interestingly, metastatic 4C11+ melanoma cell line showed higher proportion between high (around 120 kDa) and low molecular mass bands (around 105 kDa) compared to melan-a melanocytes, pre-malignant 4C cells and non-metastatic 4C11- melanoma cell line (Figure 2A). The high molecular mass band corresponds to the mature and highly glycosylated form of β1-integrin and its increase in relation to the immature form (low molecular mass) is in agreement with the literature, since β1-integrins may present aberrant glycosylation in tumorigenic cells [22].

CD63 expression, mainly on the cell surface, was evaluated in melan-a, 4C, 4C11- and 4C11+ cell lines, by flow cytometry using a specific polyclonal antibody against CD63. Higher level of CD63 protein expression was observed in pre-malignant 4C melanocytes and non-metastatic 4C11- melanoma cells compared to non-tumorigenic melan-a melanocytes and metastatic 4C11+ melanoma cells (Figure 2B).

In our melanoma model, both increase in Timp1 expression and *anoikis* resistance are acquired along tumor progression. Once it was shown that in human breast epithelial cells the interaction among CD63, TIMP1 and β1-integrin can regulate apoptosis [15], we analyzed the possible differential interaction among CD63, Timp1 and β1-integrin in cell lines corresponding to different stages of melanoma progression. For this, the interactions among CD63, Timp1 and β1-integrin proteins on the surface of melan-a, 4C, 4C11-, and 4C11+ cell lines were analyzed by protein co-immunoprecipitation assays using membrane-enriched extracts and specific antibodies for the molecules of interest. Western blot analysis performed after immunoprecipitation with anti-β1-integrin antibody showed interaction between Timp1 and β1-integrin only in the tumorigenic melanoma cell lines (4C11- and 4C11+), but not in non-tumorigenic melan-a or pre-malignant 4C melanocytes (Figure 2C).

The interaction between Timp1 and CD63 was also analyzed by immunoprecipitating CD63 complexes followed by Western blot using an antibody against Timp1. The interaction between CD63 and Timp1 was observed in pre-malignant 4C melanocytes and 4C11- and 4C11+ melanoma cell lines, but not in the parental melan-a melanocytes (Figure 2C). The analysis of membrane-enriched extracts immunoprecipitated with anti-CD63 antibody followed by Western blot using anti-β1-integrin antibody showed that β1-integrin-CD63 interaction occurs in all lineages, but is more intense along melanoma progression (4C, 4C11- and 4C11+ cell lines) (Figure 2C). Data obtained from these co-immunoprecipitation assays using cell lines representing different stages of melanocyte malignant transformation show differential interaction among CD63, Timp1 and β1-integrin along tumor progression. If the survival signaling triggered by Timp1 depends on its interaction with molecules such as CD63 and β1-integrin is still under investigation.

### Timp1 enriched medium confers clonogenic capacity and increased survival to melanocytes

**Figure 3:**
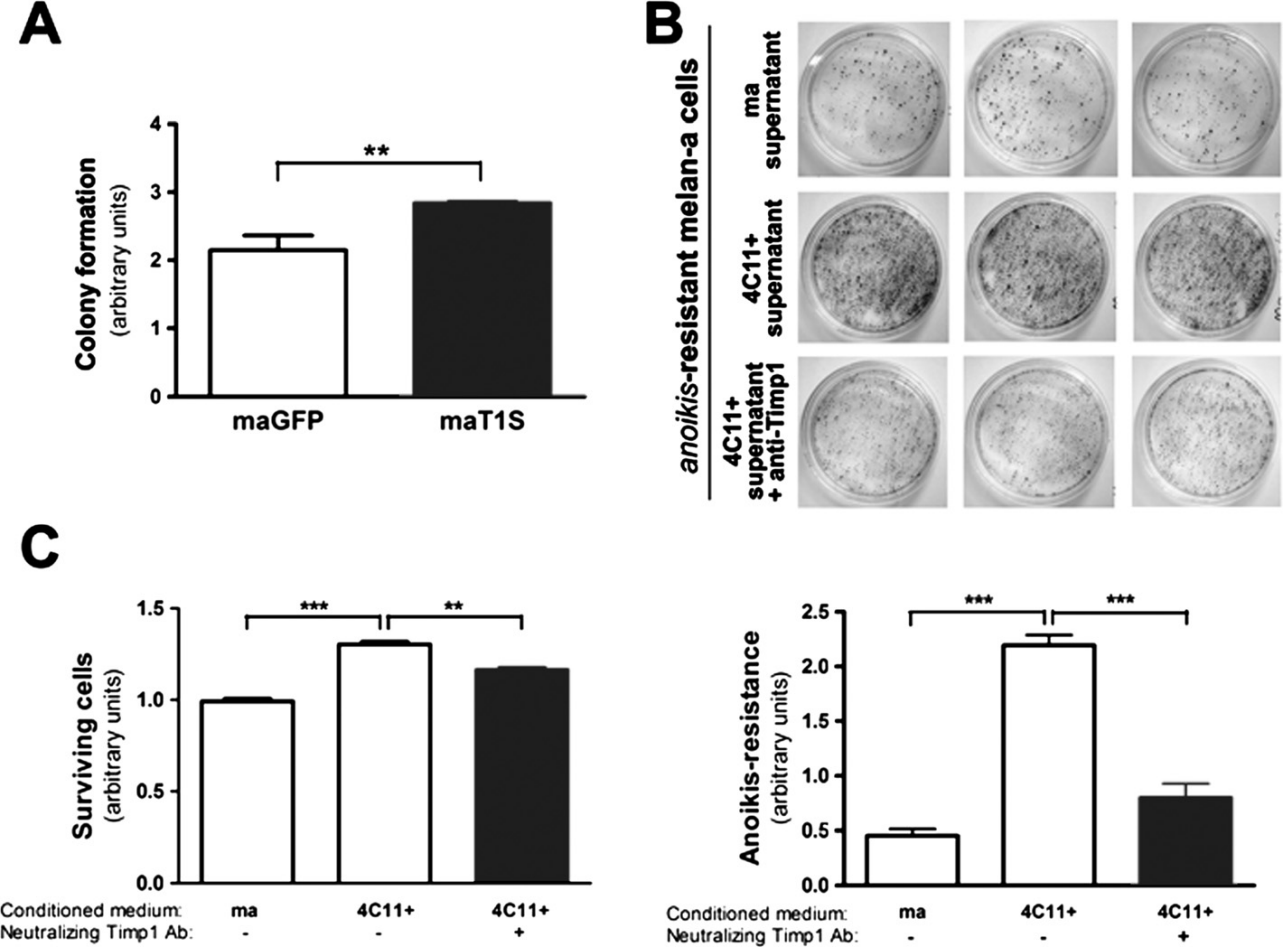
**The presence of Timp1 in the 4C11+ conditioned medium confers *anoikis *resistance to melanocytes. A.** The MaGFP and MaT1S cell lines (2x10^2^) were incubated at 37°C on plates of 60 mm^2^ adhered to the substrate for 7 days. After this period, the formation of clones was visualized by adding the dye toluidine blue in 1% borax. **B.** The melan-a melanocytes and 4C11+ melanoma cell lines were maintained in culture in RPMI pH 6.9 0.5% FCS in the presence of PMA for 48 hours. After this period, the medium conditioned by these cells was collected. Melan-a melanocytes were grown in suspension for 96 hours in three different conditions: a) with melan-a conditioned medium, b) with 4C11+ conditioned medium, or c) with 4C11+ conditioned medium in the presence of Timp1-neutralizing antibody. After 96 hours, the formation of clones by clonogenic assay was analyzed. **C.** After 96 hours of anchorage blockage, all cells were collected and viable cells were determined by MTT assay. maGFP: murine non-tumorigenic melanocyte lineage transfected with fluorescent protein GFP; maT1S: murine non-tumorigenic melanocyte overexpressing Timp1; ma: non-tumorigenic melan-a melanocytes; 4C11+: metastatic melanoma cells. **p < 0.01, ***p < 0.001.

To evaluate the effect of Timp1 overexpression in *in vitro* tumor cell traits as colony formation and survival, non-tumorigenic melan-a melanocytes overexpressing Timp1 (MaT1S) were subjected to colony formation assay. As observed in Figure 3A, Timp1-overexpressing melan-a cells (MaT1S) revealed larger potential to survive and growth under low confluence condition, resulting in an increase colony formation capability than their control, melan-a cells expressing Green Fluorescent Protein (MaGFP). Since Timp1 is present in the conditioned media of pre-malignant 4C melanocytes and 4C11- and 4C11+ melanoma cells, but not in melan-a melanocyte, and it interacts with proteins expressed on the cell surface, such as CD63 and β1-integrin, the next step was to analyze the ability of soluble Timp1 to confer *anoikis* resistance to melan-a cells. For this, two different approaches were done with melan-a melanocytes in the presence of conditioned medium from melan-a or 4C11+ cultures: the clonogenic (Figure 3B) and MTT (Figure 3C) assays after maintaining melan-a melanocytes in suspension for 96 hours. It was observed that in the presence of conditioned medium from 4C11+ melanoma cells, melan-a cells became more clonogenic than in the presence of their own conditioned medium (Figure 3B). Furthermore, the neutralization of Timp1 with a specific antibody reversed the acquisition of *anoikis*-resistant phenotype by melan-a cells in the presence of conditioned medium from 4C11+ melanoma cells (Figure 3B). The same was observed in MTT assay, in which 4C11+ conditioned medium conferred survival advantages to melan-a melanocytes during anchorage blockade and this was reversed in the presence of a Timp1-neutralizing antibody (Figure 3C). It is important to note that in this assay, all viable cells, not only adherent ones, are detected. These data indicate that exogenous soluble Timp1 confers resistance to *anoikis* to melan-a melanocytes.

### Timp1 overexpression activates PI3-K signaling pathway in melanocytes

**Figure 4:**
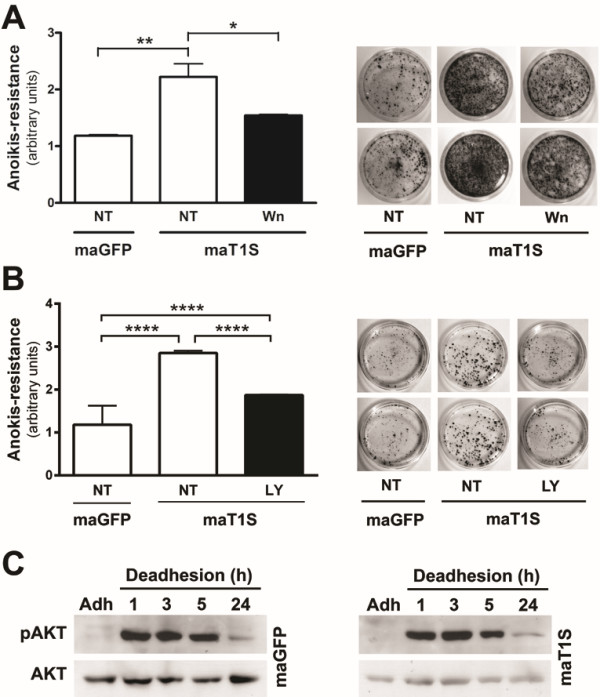
**PI3-K signaling pathway is involved in *anoikis *resistance phenotype conferred by Timp1.** The MaGFP and MaT1S cell lines were treated overnight with PI3-K inhibitors, Wortmannin (**A**) or LY294002 (**B**), and their clonogenic capability was evaluated. **C.** Melan-a melanocytes stably transfected with GFP (control transfection, MaGFP) and Timp1 (MaT1S) were maintained in suspension for 1, 3, 5 and 24 hours. The Akt activation was assessed by Western blotting. *p < 0.05, **p < 0.01, ****p < 0.0001.

As previously mentioned, several studies indicate that TIMP1 mediates activation of specific signal transduction pathways that protect cells from apoptosis in a manner that is independent of its metalloproteinase inhibitory activity [15,18]. We observed that melan-a cells that overexpress Timp1 (maT1S) were less *anoikis* resistant in the presence of Wortmannin (Figure 4A) or LY294002 (Figure 4B), indicating the involvement of PI3-K signaling pathway in this process. One of the downstream targets of PI3-K is AKT phosphorylation [18]. To investigate if Akt activation was involved in *anoikis* resistance conferred by Timp1, adherent (D0) and deadherent (D1h, D3h, D5h, D24h) MaT1S and MaGFP cells were assayed for the levels of activated Akt. Figure 4C shows that Akt is phosphorylated in melanocytes subjected to anchorage impediment; however, Timp1 overexpression did not contribute to its activation, since there was no difference between phosphorylated Akt levels in MaGFP and MaT1S cells. Our results suggest that soluble Timp1 mediates activation of PI3-K signal transduction pathways in melanocytes subjected to anchorage blockade, contributing to *anoikis* resistance, but this regulation is independent of Akt phosphorylation.

### Neutralizing Timp1 reverses *anoikis*-resistant phenotype in melanoma cells

**Figure 5:**
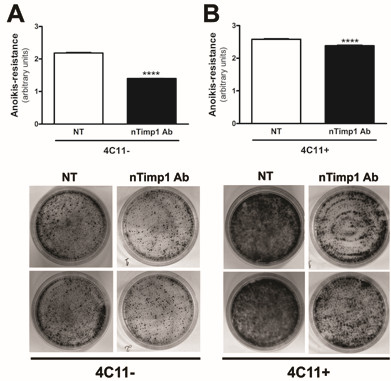
**Timp1 neutralization renders melanoma cells sensitive to *anoikis.*** Non-metastatic 4C11- (**A**) and metastatic 4C11+ (**B**) melanoma cell lines were maintained in suspension for 96 hours in the presence or absence of Timp1-neutralizing antibody. After 96 hours, suspended cells were plated and after 5 days clonogenic capacity was analyzed. 4C11-: non-metastatic melanoma cells; 4C11+: metastatic melanoma cells; NT: non-treated; nTimp1: Timp1-neutralizing antibody. ****p < 0.0001.

As previously shown, Timp1-rich conditioned medium from 4C11+ culture confers *anoikis* resistance to melan-a melanocytes. In order to check if Timp1 also confers *anoikis*-resistance in melanoma cells, non-metastatic 4C11- and metastatic 4C11+ melanoma cells were subjected to anchorage blockade for 96 hours in the presence or not of a Timp1-neutralizing antibody. As shown in Figure 5, neutralizing Timp1 renders both non-metastatic (A) and metastatic (B) cell lines more sensitive to *anoikis*.

### PI3-K inhibition abrogates survival in melanoma cells

**Figure 6:**
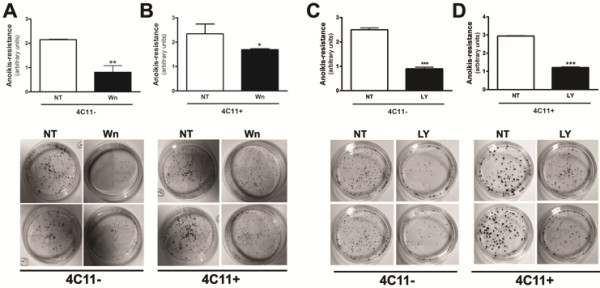
**PI3-K inhibition renders melanoma cells *anoikis *sensitive.** The 4C11- and 4C11+ melanoma cell lines were maintained in suspension for 96 hours in the presence of Wortmannin (**A** and **B**, respectively) or LY294002 (**C** and **D**, respectively). After 96 hours, suspended cells were plated and after 5 days clonogenic capacity was analyzed. 4C11-: non-metastatic melanoma cells; 4C11+: metastatic melanoma cells; NT: non-treated; Wn: Wortmannin; LY: LY294002. *p < 0.05, **p < 0.01, ***p < 0.001.

To examine whether the PI3-K signaling pathway is involved in *anoikis* resistance conferred by soluble Timp1, non-metastatic 4C11- and metastatic 4C11+ melanoma cells were maintained in suspension for 96 hours in the presence or not of Wortmannin or LY294002. After this, cells were collected and *anoikis* resistance was analyzed. Figure 6 shows that both Wortmannin and LY294002 sensitized non-metastatic 4C11- (A and B, respectively) and metastatic 4C11+ (C and D, respectively) melanoma cells to *anoikis*. These data suggest that PI3- signaling pathway activation confers *anoikis* resistance to melanoma cells that overexpress Timp1.

### TIMP1 is superexpressed and associated with β1-integrins in human metastatic melanoma cells, but not in primary human melanocytes

**Figure 7:**
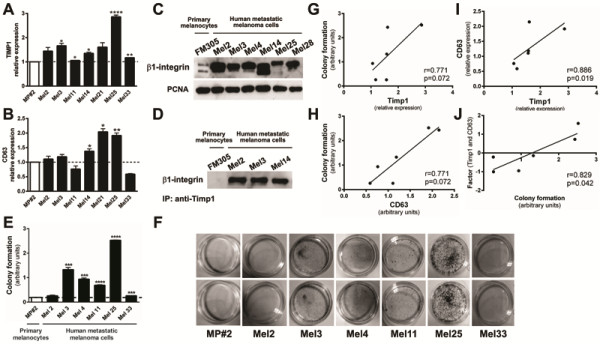
**Increased levels of *TIMP1, CD63 *and β1-integrins in melanoma cells compared to primary melanocytes and association between Timp1 and β1- integrins only in human metastatic melanoma cells.** mRNA levels of *TIMP1* and *CD63* were measured by real time PCR (**A** and **B**, respectively). **C.** β1-integrin expression was analyzed in primary human melanocytes MP#2 and human metastatic melanoma cell lines (Mel2, Mel3, Mel4, Mel14, Mel25 and Mel28) by Western blotting using a specific polyclonal antibody. The same membrane was reprobed with anti-PCNA antibody as an internal control. **D.** Membrane-enriched protein extracts from MP#2, Mel2, Mel3 and Mel14 cell lines were immunoprecipitated with anti-TIMP1 and analyzed by Western blotting with anti-β1-integrin. **E** and **F.** Primary human MP#2 melanocytes and human metastatic melanomas Mel2, Mel3, Mel4, Mel11, Mel25, Mel28 and Mel33 (2x10^2^) were incubated at 37°C on 60 mm plates and colony formation capacity was determined after 5 days. Spearman’s correlation (r) was used to correlate *TIMP1* and clonogenic capacity (**G**), *CD63* expression and clonogenic capacity (**H**), *TIMP1* and *CD63* expression (**I**), and combined *TIMP1* and *CD63* expression and clonogenic capacity (**J**). All analyses were performed using SPSS (v 18, Chicago, IL, USA). The significance level was set at 0.05.

TIMP1 levels are increased in cancer patients, particularly in those with breast or colorectal carcinoma, and this augment is negatively associated with patient outcome [23]. Recent studies have suggested the clinical utility of TIMP1 as a biomarker and independent prognostic factor in breast, colorectal and several hematological cancers. They are consistent with the anti-apoptotic activity of TIMP1 mediated by CD63/ β1-integrins binding. Increased transcript level of Timp1 and CD63 was shown in different human metastatic melanoma cell lines compared to primary human melanocytes (Figure 7, A and B). Five from seven metastatic melanoma cells expressed significantly higher levels of *TIMP1* (Figure 7A), and three from seven melanoma cell lines presented increased levels of *CD63* (Figure 7B) compared to primary human melanocytes. All of six metastatic melanoma cell lines analyzed showed increased expression of β1-integrins (Figure 7C), and all cell lines, except Mel14, presented increased ratio between high and low molecular mass β1-integrin. The association between β1-integrins and TIMP1 was observed in the human metastatic melanoma cells, but not in primary melanocytes (Figure 7D). All metastatic melanoma cell lines studied have more colony formation capacity when compared to primary melanocytes (Figure 7E). Interestingly, we found a moderate correlation between the expression levels of *TIMP1* and *CD63* and ability to form colonies (Figure 7G and 7H, respectively). A strong correlation was found between the expression of *TIMP1* and *CD63* (Figure 7I) and, when these two variables were combined in a factor, they strongly correlate with colony formation capacity (Figure 7J), reinforcing the possible role of *TIMP1*, mainly when associated with increased expression of *CD63*, in melanoma genesis.

## Discussion

Previous data from our laboratory showed a significant increase in Timp1 expression along melanocyte malignant transformation, in parallel with the acquisition of an *anoikis*-resistant phenotype [17]. Increasing Timp1 expression rendered melanoma cell more *anoikis*-resistant and more efficient in metastases development, indicating the correlation between Timp1 and poor prognosis in melanoma [17]. Indeed, different studies have correlated increased TIMP1 expression with malignant progression and poor prognosis, both in human tumors and murine experimental models [12,24,25]. It has been commonly shown that TIMP1 is involved in the regulation of proliferation, cell survival, and differentiation of numerous cell types [26,27], angiogenesis [28,29] and apoptosis [13,30] through mechanisms apparently independent of MMPs. In this regard, Liu and colleagues showed that TIMP1 expression protects breast epithelial cells and human lymphocytes to different stimuli that induce apoptosis [31]. In addition, Kluger and colleagues identified TIMP1 as a plasma marker in patients with metastatic melanoma [32].

Melanocytes overexpressing Timp1 acquired *anoikis*-resistant phenotype [17] and showed more colony formation capability than control MaGFP (Figure 3A), reinforcing that Timp1 can regulate cell survival in melanocytes. TIMP1 functions are determined depending on its level and localization (soluble *vs*. pericellular). TIMP1 is a secreted protein and may associate with cell surface and extracellular matrix in different cell types where it can interact with its partners and mediate cellular processes [15,23]. In our model, Timp1 was found in conditioned medium from all cell lines corresponding to different steps of melanoma progression (4C, 4C11- and 4C11+), except in melan-a melanocytes (Figure 1C). Although *timp1* transcript is increased in metastatic 4C11+ melanoma cell line compared to pre-malignant 4C and non-metastatic 4C11- melanoma cell line (Figure 1A) and Timp1 protein is augmented at cell surface since pre-malignant 4C to metastatic 4C11+ cell line (Figure 1B), its presence in the supernatant is more expressive in pre-malignant 4C and non-metastatic 4C11- cells compared to melan-a melanocytes (Figure 1C). The explanation of this discrepancy is still under investigation. Despite the increase in Timp1 expression in 4C11- and 4C11+ melanoma cell lines, high MMP activity was found in these melanoma cell lines (Figure 1D), suggesting MMP-independent functions of Timp1. Recently, Kim and coworkers showed that colon cancer cells have TIMP1 molecule bearing aberrant glycosylation, which impairs its efficient function as MMP inhibitor [33]. In fact, an increase in *N-*linked oligosaccharides was observed in melanoma cells derived from melan-a subjected to sequential cycles of deadhesion [10], reinforcing the idea that TIMP1 would confer tumor aggressiveness independent of its function on MMP activity. Several studies have shown changes in the expression pattern of integrins in different malignant tumors [34,35]. Besides that, alterations in integrin distribution on cell surface and in post-translational modifications were also observed in many tumor types [35].

Several authors showed association between TIMPs and integrins [15,36]. Binding studies have shown that TIMP2 interacts with α3β1 integrin on the cell surface in human endothelial cells [37]. Interaction between TIMP1 and αvβ3 confers protection against apoptosis induced by TNF-α In human osteosarcoma cell lines [37].

Tetraspanins were also described as binding partners for TIMPs [37]. Tetraspanin family members, including CD63, CD82 and CD151, also interact with adhesion molecules, as integrins, and modulate transduction pathways that regulate adhesion, motility and survival [38]. There are reports indicating that CD63 can regulate both positively and negatively integrin activity [38]. Several studies reported alteration in CD63 expression along melanoma progression [2,39,40]. In our study, the presence of CD63 protein was observed in all cell lines, especially in non-metastatic 4C11- melanoma cells (Figure 2B). This result is in agreement with published data that showed increased CD63 expression mainly in early stages of melanoma [41]. Furthermore, metastatic 4C11+ melanoma cells present decreased CD63 expression compared to non-metastatic 4C11-melanoma cells (Figure 2B), corroborating the data showing that inhibition of CD63 expression increased cell motility, matrix degrading activity and invasiveness of melanoma cells *in vitro*[42]. Recently, CD63 protein was detected in human breast epithelial cells as a binding partner of TIMP1 on cell surface [15]. These authors confirmed by immunoprecipitation and confocal microscopy the co-localization of CD63 and TIMP1 with β1-integrin and showed that reduced levels of CD63 by shRNA resulted in reduced binding of TIMP1 to cell surface, β1-integrin activation and cell survival signaling of breast epithelial cells. Other authors have also shown that CD63 regulates some signaling pathways involved in anti-apoptotic activity of TIMP1 [43].

**Figure 8:**
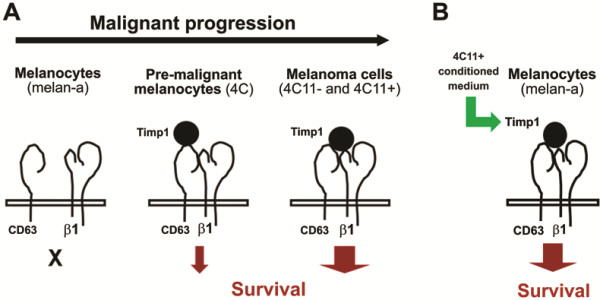
**Hypothesis for the assembly of CD63/β1-integrins/Timp1 complex along melanoma genesis.** In the absence of Timp1, almost no association between CD63 and β1-integrins occurs in melan-a melanocytes, which might contribute to their *anoikis* sensitivity. The presence of these molecules in pre-malignant 4C melanocytes and 4C11- and 4C11+ melanoma cells result in the formation of CD63/β1-integrins/Timp1 complex and acquisition of *anoikis*-resistant phenotype. The absence of physical interaction between Timp1 and β1-integrins might result in a less efficient survival transduction pathway, differently of what is seen in melanoma cell lines, in which this complex seems to be more tightly assembled.

In our model, CD63 is interacting with Timp1 in pre-malignant 4C melanocytes and in tumorigenic cell lines (Figure 2C), cells that acquire an *anoikis*-resistant phenotype (Figure 1E). In these cells, CD63 may be recruiting signaling molecules responsible for the increase in cell survival, since the short cytoplasmic tail of CD63 interacts with signaling molecules including phosIphatidylinositol 4-kinase (PI4-K) and Src [44-47] and regulates signaling pathways involving proteins such as FAK, Gab2, PI3-K and Akt [48-50]. However, we did not observe interaction between β1-integrin and Timp1 in pre-malignant 4C cell line. Timp1 was shown to interact with β1-integrins only in 4C11- and 4C11+ melanoma cells (Figure 2C). Therefore, it is possible to suggest that, in this model, the tight complex comprised by Timp1, CD63 and β1-integrins contributes to the acquisition of malignant phenotype, since the interaction among these three molecules was observed only in melanoma cell lines (Figure 8). Preventing adhesion to the substrate could result in changes in the structure and composition of lipid rafts, glycosphingolipid-cholesterol-enriched microdomains, affecting Timp1/CD63/β1-integrin complex assembly along melanoma development. The CD63 tetraspanin and β1-integrin may be found in lipid rafts, where CD63 can recruit signaling proteins [48]. Furthermore, alterations in integrin conformation control their capability to bind to ligands [49]. However, it has been demonstrated that a significant proportion of complexes containing integrins and tetraspanins are compartmentalized out of lipids rafts [50-52]. It is also possible that integrin-tetraspanin complexes shuttle in and out of rafts and thereby control the recruitment of different proteins into these compartments, consequently modulating signaling events in many ways [50]. In this way, the sequential cycles of anchorage blockade may alter this compartmentalization and lead to alterations in signaling pathways, resulting in malignant transformation. Next, we evaluated the impact of secreted Timp1 on melanocytes cell survival. As shown in Figure 3B and C, non-tumorigenic melan-a melanocytes maintained in suspension for 96 hours in the presence of conditioned medium from 4C11+ melanoma cells were more *anoikis*-resistant than those maintained in the presence of conditioned medium from melan-a themselves. Moreover, increased *anoikis* resistance conferred by conditioned medium from 4C11+ melanoma cells was abrogated by the presence of Timp1-neutralizing antibody, demonstrating that extracellular Timp1 is important to render melan-a melanocytes *anoikis*-resistant.

Interaction between Timp1 and CD63 might activate survival signaling pathways mediated by β1-integrins, including FAK and Src, resulting in PI3-K signaling pathway activation [15,46]. In our model, the use of PI3-K signaling pathway inhibitors sensitized Timp1-overexpressing melanocytes to *anoikis* (Figure 4A and B), suggesting the involvement of PI3-K signal transduction in regulation of cell survival by Timp1. However, Timp1 did not change Akt activation, since melan-a cells overexpressing Timp1 showed the same levels f phospho-Akt compared to control melan-a cells (Figure 4C). Indeed, recent studies have shown that PI3-K signaling pathway can act independently of AKT phosphorylation, by activating PDK1, which in turn activates other proteins such as PKC [53,54]. It is possible that PKC is constitutively activated in melanoma cells since their survival is PMA independent [10]. Moreover, both PI3-K inhibitor and Timp1-neutralizing antibody decreased *anoikis* resistance of melanoma cells (Figure 6), suggesting that also in tumorigenic cells survival modulation by Timp1 is regulated by PI3-K signaling pathway.

Several studies pointed TIMP1 as a promising tumor biomarker, since it has been found in high levels in the plasma of patients bearing different types of cancer. Clinical studies using primary breast tumors, for example, have shown an association between high levels of TIMP1 mRNA and protein and poor prognosis in patients with breast cancer [55,56]. Moreover, high levels of TIMP1 in tumor tissue are strongly associated with poor response to chemotherapy [57,58]. In addition, we observed high levels of TIMP1 in five from seven metastatic human melanoma cell lines analyzed (Figure 7A). Both *TIMP1* and *CD63* levels correlated with colony formation capacity (Figure 7G and H). A strong and significant correlation was found between *TIMP1* and *CD63* levels (Figure 7I) and, more interestingly, *TIMP1* and *CD63* expression combined in a factor strongly correlates to colony formation ability (Figure 7J), pointing TIMP1, mainly when associated with increased CD63 expression, as a potential biomarker of melanoma.

The absence of CD63/β1-integrins/Timp1 complex formation in the parental non-tumorigenic melan-a melanocytes may be the responsible for the lack of a survival signaling to prevent *anoikis*. In the case of cell line corresponding to pre-malignant melanocytes (4C), interactions between CD63/β1-integrins and CD63/Timp1 were observed, which could initiate PI3-K signaling pathway for cell survival, since the 4C cell line is more resistant to *anoikis* when compared with its parental lineage, melan-a. CD63/β1-integrins, CD63/Timp1 and Timp1/β1-integrins interactions were detected in the tumorigenic 4C11- and 4C11+ cell lines, suggesting that CD63/β1-integrins/Timp1 form a tighter complex when compared to that in pre-malignant melanocytes (4C), which could result in more efficient activation of PI3-K signaling pathway and in melanoma development (Figure 8), indicating the relevance of supramolecular complex formation in tumor progression.

In conclusion, our data demonstrate differential interaction among CD63, Timp1 and β1-integrins between normal and melanoma cells and that this binding may regulate essential processes for tumorigenesis such as *anoikis* resistance. These findings provide evidences that the supramolecular complex containing Timp1, CD63 and β1- integrin triggers PI3-K signaling pathway that contribute to melanoma progression through apoptosis inhibition (Figure 8). A better understanding of how Timp1 modulates *anoikis* resistance during the melanoma progression may be valuable in developing new therapeutic interventions.

## Abbreviations

PMA: Phorbol 12-myristate 13-acetate; Timp: Tissue inhibitor of Metalloproteinases; MMPs: Metalloproteinases; ECM: Extracellular matrix; PI3-K: phosphoinositide 3-kinase.

## Author contributions

Conceived and designed the experiments: MT, FHMM, MGJ. Performed the experiments: MT, GBP. Analyzed the data: MT, FHMM, GBP, MGJ. Contributed reagents/materials/analysis: MT, FHMM, GBP, DCPS, MGJ. Wrote the paper: MT, FHMM, MGJ. All authors read and approved the final manuscript.
