# Erratum: Timp1 interacts with beta-1 integrin and CD63 along melanoma genesis and confers anoikis resistance by activating PI3-K signaling pathway independently of Akt phosphorylation

**DOI:** 10.1186/s12943-015-0405-2

**Published:** 2015-08-22

**Authors:** Mariana Toricelli, Fabiana H. M. Melo, Giovani B. Peres, Débora C. P. Silva, Miriam G. Jasiulionis

**Affiliations:** Pharmacology Department, Universidade Federal de São Paulo, São Paulo, Brazil; Microbiology, Immunology and Parasitology Department, Universidade Federal de São Paulo, São Paulo, Brazil; Biochemistry Department, Universidade Federal de São Paulo, São Paulo, Brazil; Ludwig Institute for Cancer Research, São Paulo, Brazil

## Erratum

After publication of this study [1], we found out that we unfortunately sent two figures in duplicate. They are Fig. 4b NT and Fig. 6c NT [1]. It is important to emphasize that the results shown in the graphs are correct since they represent the mean of three independent biological assays, each of them made in technical triplicates. The photographs are only representative figures of three biological assays.

**Fig. 4:**
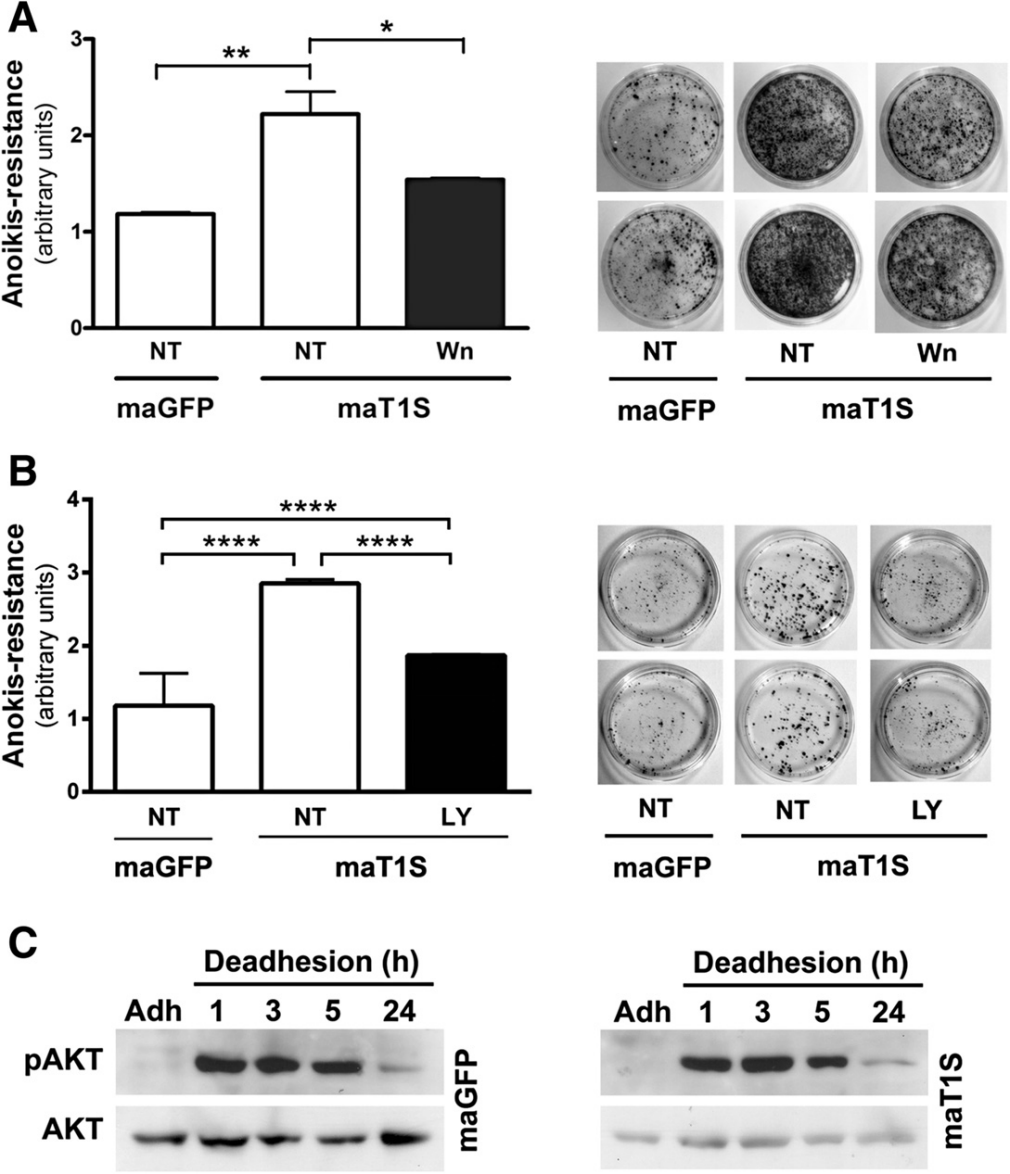
PI3-K signaling pathway is involved in *anoikis* resistance phenotype conferred by Timp1. The MaGFP and MaT1S cell lines were treated overnight with PI3-K inhibitors, Wortmannin (**a**) or LY294002 (**b**), and their clonogenic capability was evaluated. **c** Melan-a melanocytes stably transfected with GFP (control transfection, MaGFP) and Timp1 (MaT1S) were maintained in suspension for 1, 3, 5 and 24 hours. The Akt activation was assessed by Western blotting. *p < 0.05, **p < 0.01, ****p < 0.0001

**Fig. 6:**
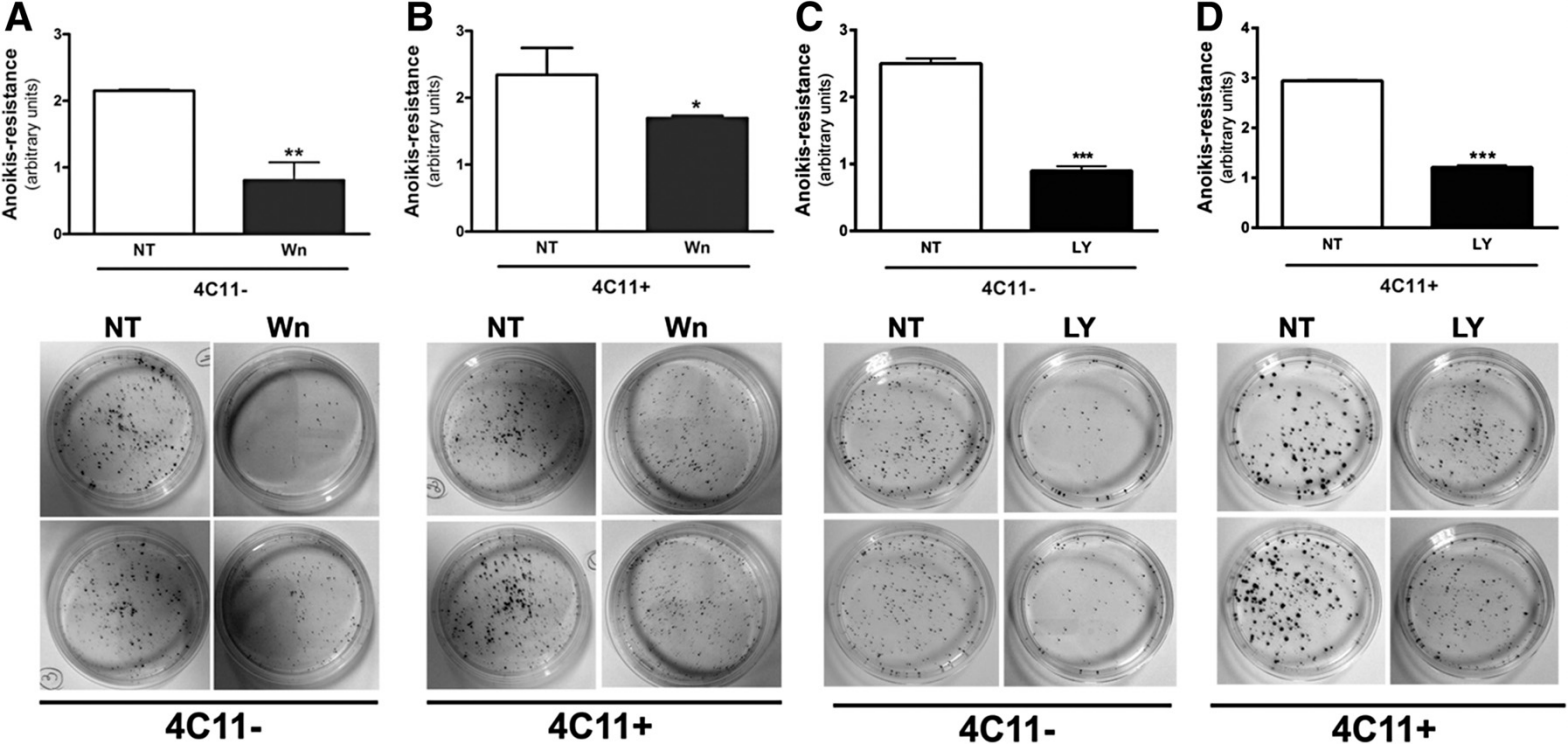
PI3-K inhibition renders melanoma cells *anoikis* sensitive. The 4C11- and 4C11+ melanoma cell lines were maintained in suspension for 96 hours in the presence of Wortmannin (**a** and **b**, respectively) or LY294002 (**c** and **d**, respectively). After 96 hours, suspended cells were plated and after 5 days clonogenic capacity was analyzed. 4C11-: non-metastatic melanoma cells; 4C11+: metastatic melanoma cells; NT: non-treated; Wn: Wortmannin; LY: LY294002. *p < 0.05, **p < 0.01, ***p < 0.001
